# Independent and combined associations of diabetes and thyroid disorders with risks of incident dementia

**DOI:** 10.3389/fendo.2026.1874555

**Published:** 2026-07-24

**Authors:** Jing Guo, Jiayu Li, Bin Gao, Ke Liu, Xiaohui Sun, Ding Ye, Yingying Mao

**Affiliations:** 1 School of Public Health, Zhejiang Chinese Medical University, Hangzhou, Zhejiang, China; 2 Department of Psychiatry, The Second Affiliated Hospital, Zhejiang University School of Medicine, Hangzhou, Zhejiang, China

**Keywords:** cohort study, dementia, diabetes, epidemiology, thyroid disorders

## Abstract

**Background:**

Diabetes and thyroid disorders are modifiable risk factors for cognitive impairment. However, no studies have been conducted to comprehensively examine associations of different types of the two diseases, as well as their combinations, with dementia risks.

**Methods:**

Based on the data from UK Biobank study, patients with different types of diabetes and thyroid disorders were identified with the hospital inpatient record. Status of dementia was assessed with multi-source information. Independent and combined associations of diabetes and thyroid disorders with dementia were estimated with Cox regression models.

**Results:**

Among 284,081 participants, 8,236 incident cases of all-cause dementia (ACD) [3,755 Alzheimer’s disease (AD) cases, 1,893 vascular dementia (VD) cases] were identified over a mean [standard deviation (SD)] follow-up of 13.03 (2.29) years. Four types of diabetes and two types of thyroid disorders were significantly associated with increased risks of dementia. Compared with participants without diabetes and thyroid disorders, those with only thyroid disorders [hazard ratio (HR) = 1.33, 95% confidence interval (CI) = 1.21 to 1.47, *P* < 0.001], only diabetes (HR = 1.76, 95% CI = 1.64 to 1.89, *P* < 0.001), and both (HR = 2.16, 95% CI = 1.84 to 2.53, *P* < 0.001) had elevated risks of ACD (*P*_trend_ < 0.001), as well as higher risks of AD and VD. Combined associations of hypothyroid and diabetes and of hyperthyroid and diabetes were also significantly positive (all *P* values < 0.05). Moreover, cumulative years of diabetes and thyroid were related to higher risks of dementia.

**Conclusions:**

Diabetes, thyroid disorders, and their combinations were associated with increased risks of dementia in adults aged 55 years and older.

## Highlights

- Different types of diabetes and thyroid diabetes were associated with dementia.
- Combinations of diabetes and thyroid diabetes were associated with dementia.
- Cumulative years of diabetes and thyroid diabetes were associated with dementia.

## 1 Introduction

Current evidence indicated that there were 57 million people living with dementia worldwide in 2019 and the number of cases is expected to be three times higher by 2050 (1). The disease burden of dementia is heavy to the individuals, caregivers, and society, with global costs of about one trillion dollars annually (2). Alzheimer’s disease (AD, 60% to 80%) and vascular dementia (VD, 5% to 10%) were the most common types of dementia (3). AD is characterized by the accumulation of neurofibrillary tangles and amyloid plaques in the brain, inducing progressive neurodegeneration; VD is caused by the cerebrovascular insults linked to reduced blood flow to the brain, leading to neuronal injury and cognitive decline (4). Due to the lack of effective medical treatments, the primary prevention through modifiable risk factors has been proposed as an urgent priority to reduce the incidence of dementia (2).

Diabetes and thyroid disorders, the two most common endocrine dysfunctions, often coexist in the population (5). Diabetes is a heterogeneous metabolic disorder characterized by hyperglycaemia due to insulin resistance (in the case of type 2 diabetes) or insufficient insulin secretion (in type 1 diabetes) (6). Recent studies strongly suggest associations of diabetes with biomarkers of AD (7) and elevated risks of cognitive impairment and dementia (8). Diabetes is regarded as a potentially modifiable risk factor for dementia (2). Most previous studies focused on associations between type 2 diabetes or mixed diabetes and dementia (8), however, fewer examined the associations of type 1 diabetes (9, 10). Besides, dementia risks in association with type 1 diabetes were not adjusted for educational levels or lifestyles which play important roles in dementia occurrence in these previous studies (9, 10).

Thyroid disease is mainly characterized by abnormalities in the function or structure of thyroid gland, such as increased (hyperthyroidism) or decreased (hypothyroidism) plasma levels of thyroid hormone, as well as enlargement of the gland (goiter or single thyroid nodule) (11). As a potentially reversible cause of cognitive decline, thyroid dysfunction is frequently accompanied with dementia (12). Thyroid function test is proposed as an essential component of the workup for the dementia diagnosis (13). However, effects of thyroid disorders on dementia have not been elucidated due to inconsistent findings. Hypothyroidism links to increased risks of dementia in the cross-sectional study of Asians (14) and the cohort study of Europeans (15). People with hyperthyroidism were also found to have higher risks of cognitive disorders (16) and incident dementia in the U.S. cohort studies (17). However, data of a multicohort study from different countries indicated that hypothyroidism and hyperthyroidism were not associated with cognitive function, cognitive decline, or dementia (13).

To the best of our knowledge, no studies have been performed to consider multiple types of diabetes (e.g., type 1 and type 2 diabetes) and thyroid disorders (e.g., hyperthyroidism, hypothyroidism, and autoimmune thyroiditis) simultaneously within a single cohort. Besides, combined effects of the comorbidity of diabetes and thyroid disease on dementia risks have not been examined previously. Based on the data from UK Biobank study, we aimed to comprehensively analyze associations of multiple types of diabetes and thyroid disorders, as well as their combinations, with the incidence of dementia.

## 2 Methods

### 2.1 Study design and participants

The UK Biobank study is an ongoing population-based cohort which recruited more than 500,000 participants aged 40 to 70 years at 22 assessment centers across the UK (18). Baseline data were collected between 2006 and 2010, covering a wide range of topics, including demographic characteristics, lifestyle, health status, and economic condition through extensive questionnaires, interviews, and physical examinations. Clinical outcomes of participants have been followed via hospital inpatient records, death certificates, and primary care records. The UK Biobank study was approved by the National Health Service North West Multicenter Research Ethics Committee. All participants provided the written informed consent.

In the present study, participants were restricted among those with data on hospital inpatient diagnosis [International Classification of Diseases 10^th^ Revision (ICD-10)]. Among 502,355 participants at baseline, we excluded individuals who withdrew their consent during the follow up (n = 116), who were younger than 55 years at baseline (since the majority of dementia cases occur in the late life, n = 194,033), who had prevalent dementia at baseline (n = 215), or who had missing data on the covariates (n = 23,910) (**Supplementary Figure 1**). A total of 284,081 participants retained for the data analysis, of whom 8,263 developed an incident dementia.

### 2.2 Assessment on diabetes and thyroid disorders

According to the ICD codes from the hospital inpatient data, diagnoses of diabetes (Fields 41270 and 41280, ICD-10: E10, E11, E12, E13, E14, and E891) and thyroid disorders (Fields 41270 and 41280, ICD-10: E00, E01, E02, E03, E04, E05, E062, E063, E065, E07, E350, E890) were ascertained (**Supplementary Table 1**). We dropped the diseases which had numbers of cases less than 100 (E01, E02, E062, E065, E12, and E891). As a result, four types of diabetes [insulin-dependent diabetes mellitus (E10), non-insulin-dependent diabetes mellitus (E11), other specified diabetes mellitus (E13), and unspecified diabetes mellitus (E14)] and six types of thyroid disorders [other hypothyroidism (E03), other nontoxic goitre (E04), thyrotoxicosis (hyperthyroidism, E05), autoimmune thyroiditis (E063), other disorders of thyroid (E07), and postprocedural hypothyroidism (E890)] were used for data analysis.

### 2.3 Assessment on dementia

The UK Biobank Outcome Adjudication Group and clinical experts have developed and validated algorithms based on the multiple sources of data including self-report, hospital admission, primary care, and death register record to ascertain a range of health outcomes. All-cause dementia (ACD), AD, and VD were extracted from the algorithmically-defined outcomes (UK Biobank Category 47) and first occurrences of health outcomes (UK Biobank Category 2405, 2406) (**Supplementary Table 1**). The method of dementia identification in the UK Biobank has also been applied in previous studies (19, 20).

### 2.4 Covariates

Covariates included age (in years), sex (male, female), race or ethnicity (White, Asian or Asian British, Black or Black British, mixed, and others), educational levels (higher, upper secondary, lower secondary, vocational, and others), smoking (current, never, previous), drinking (< 1 drink per month, 1 to 3 drinks per month, 1 to 4 drinks per week, daily or almost daily drinking), diet (unhealthy, healthy), regular physical activity (yes, no), body mass index (BMI) categories (underweight, normal weight, overweight, obese), allele status of apolipoprotein E (*APOE*) ϵ4 (noncarrier, carrier), history of self-reported diseases (no, yes) of heart diseases, stroke, hypertension, and emphysema or chronic bronchitis. Levels of education were categorized into higher (university or professional qualification), upper secondary (second/final stage of secondary education), lower secondary (first stage of secondary education), vocational (work-related qualification), and others (21). Healthy diet was defined as the adequate intake of at least four of seven ideal dietary components for cardiovascular health (high levels of fruits, vegetables, fish, and whole grain, and low levels of processed meat, unprocessed red meat, and refined grains) (21). Regular physical activity was defined as attending at least 150 minutes of moderate activity or 75 minutes of vigorous activity per week, or engaging in moderate activity at least five days or one day of vigorous activity per week (21). *APOE*-ϵ4 carriers were those who had at least one ϵ4 allele.

### 2.5 Statistical analysis

Baseline characteristics between the nondemented participants and those who developed an incident dementia were compared with the t-test and Chi-square test for continuous and categorical variables, respectively. Cox regression models were applied to examine associations of diabetes and thyroid disorders with risks of incident ACD, AD, and VD. Model 1 was adjusted for age, sex, race or ethnicity, and educational levels. Model 2 was additionally adjusted for covariates of smoking, drinking, diet, physical activity, category of body mass index, allele status of *APOE*-ϵ4, and self-reported histories of heart diseases, stroke, hypertension, and emphysema or chronic bronchitis. Follow-up time was calculated as time intervals between baseline and the date of the first diagnosis of dementia, loss to follow-up, death, or the last date of hospital inpatient admission (31 October 2022 for England, 31 August 2022 for Scotland, and 31 May 2022 for Wales), whichever occurred first.

We firstly analyzed the independent associations of a specific type of diabetes or thyroid disorders with dementia. The method of false discovery rate (FDR) was applied to adjust for *P* values of association results in consideration of multiple comparisons (**Supplementary Table 2**). Four types of diabetes (E10, E11, E13, and E14) and two types of thyroid disorders (E03, and E05) with significant independent associations (FDR-adjusted *P* values < 0.05) were extracted to assess the combined associations. The overall diabetes was defined as the occurrence of any of the four selected types of diabetes, and the same for the definition of overall thyroid disorders. Participants were categorized into four groups of individuals without diabetes and thyroid disorders (reference group), with only thyroid disorders, with only diabetes, and with both to estimate the combined effects on dementia. Similar methods were used for combinations of hypothyroidism (E03) and diabetes, and of hyperthyroidism (E05) and diabetes.

Cumulative years of diabetes and thyroid disorders were separately calculated as the years between the first occurrence of a given disease and the baseline. Total of cumulative years of diabetes and thyroid disorders was calculated as the sum of the two diseases. Associations between the cumulative years of the two diseases and incident dementia have also been estimated.

## 3 Results

### 3.1 Baseline characteristics

As shown in **Table 1**, there were 132,271 males and 151,810 females with a mean (SD) age of 62.03 (4.08) years among 284,081 participants. The majority of participants were White (n = 275,445), accounting for 96.96%. A total of 8,263 incident cases of ACD (including 3,755 AD cases and 1,893 VD cases) were identified over a mean (SD) follow-up of 13.03 (2.29) years, for a total of 6,635,896 person-years. Compared with nondemented participants, those who developed an incident dementia were more likely to be older, males, previous smoking, less drinking, obese, and *APOE*-ϵ4 carriers. Individuals with an incident dementia were also more likely to have lower levels of education and higher proportions of self-reported diseases.

**Table 1:** Baseline characteristics between participants with and without an incident dementia.

Characteristics	Total (n = 284081)	Incident (n = 8263)	Nondemented (n = 275818)	*P* value
Age (years), mean (SD)	62.03 (4.08)	64.90 (3.50)	61.94 (4.06)	< 0.001
Sex, n (%)				< 0.001
Male	132271 (46.56)	4305 (52.10)	127966 (46.40)	
Female	151810 (53.44)	3958 (47.90)	147852 (53.60)	
Race/ethnicity, n (%)				0.004
White	275445 (96.96)	7991 (96.71)	267454 (96.97)	
Asian or Asian British	3376 (1.19)	98 (1.19)	3278 (1.19)	
Black or Black British	2234 (0.79)	95 (1.15)	2139 (0.78)	
Mixed	934 (0.33)	26 (0.31)	908 (0.33)	
Others	2092 (0.74)	53 (0.64)	2039 (0.74)	
Education, n (%) ^a^				< 0.001
Higher	128760 (45.33)	2910 (35.22)	125850 (45.63)	
Upper secondary	17703 (6.23)	467 (5.65)	17236 (6.25)	
Lower secondary	54960 (19.35)	1478 (17.89)	53482 (19.39)	
Vocational	18401 (6.48)	559 (6.77)	17842 (6.47)	
Others	64257 (22.62)	2849 (34.48)	61408 (22.26)	
Smoking, n (%)				< 0.001
Current	24642 (8.67)	792 (9.58)	23850 (8.65)	
Never	145765 (51.31)	3830 (46.35)	141935 (51.46)	
Previous	113674 (40.01)	3641 (44.06)	110033 (39.89)	
Drinking, n (%)				< 0.001
Less than 1 drink per month	55135 (19.41)	2183 (26.42)	52952 (19.20)	
1 to 3 drinks per month	28701 (10.10)	826 (10.00)	27875 (10.11)	
1 to 4 drinks per week	134271 (47.27)	3514 (42.53)	130757 (47.41)	
Daily or almost daily drinking	65974 (23.22)	1740 (21.06)	64234 (23.29)	
Diet, n (%)				0.558
Unhealthy	183275 (64.52)	5356 (64.82)	177919 (64.51)	
Healthy	100806 (35.48)	2907 (35.18)	97899 (35.49)	
Regular physical activity, n (%) ^c^				< 0.001
No	79043 (27.82)	2516 (30.45)	76527 (27.75)	
Yes	205038 (72.18)	5747 (69.55)	199291 (72.25)	
BMI categories, n (%)				< 0.001
Underweight (< 18.5 kg/m^2^)	1321 (0.47)	55 (0.67)	1266 (0.46)	
Normal (≥ 18.5 to < 25 kg/m^2^)	86777 (30.55)	2449 (29.64)	84328 (30.57)	
Overweight (≥ 25 to < 30 kg/m^2^)	126074 (44.38)	3616 (43.76)	122458 (44.40)	
Obese (≥ 30 kg/m^2^)	69909 (24.61)	2143 (25.93)	67766 (24.57)	
*APOE*-ϵ4, n (%)				< 0.001
Noncarrier	203944 (71.79)	3800 (45.99)	200144 (72.56)	
Carrier	80137 (28.21)	4463 (54.01)	75674 (27.44)	
History of self-reported diseases, n (%)				
Heart diseases	17925 (6.32)	1060 (12.87)	16865 (6.12)	< 0.001
Stroke	5565 (1.96)	408 (4.95)	5157 (1.87)	< 0.001
Hypertension	176673 (62.19)	5949 (72.00)	170724 (61.90)	< 0.001
Emphysema/chronic bronchitis	5916 (2.09)	293 (3.55)	5623 (2.04)	< 0.001
Follow-up time (year), mean (SD)	13.03 (2.29)	9.92 (3.10)	13.13 (2.19)	< 0.001

*APOE*, apolipoprotein E; BMI, body mass index; SD, standard deviation.

^a^ Levels of education were categorized into higher (university or professional qualification), upper secondary (second/final stage of secondary education), lower secondary (first stage of secondary education), vocational (work-related qualification), and others.

### 3.2 Independent associations of diabetes and thyroid disorders with risks of dementia

**Figure 1:**
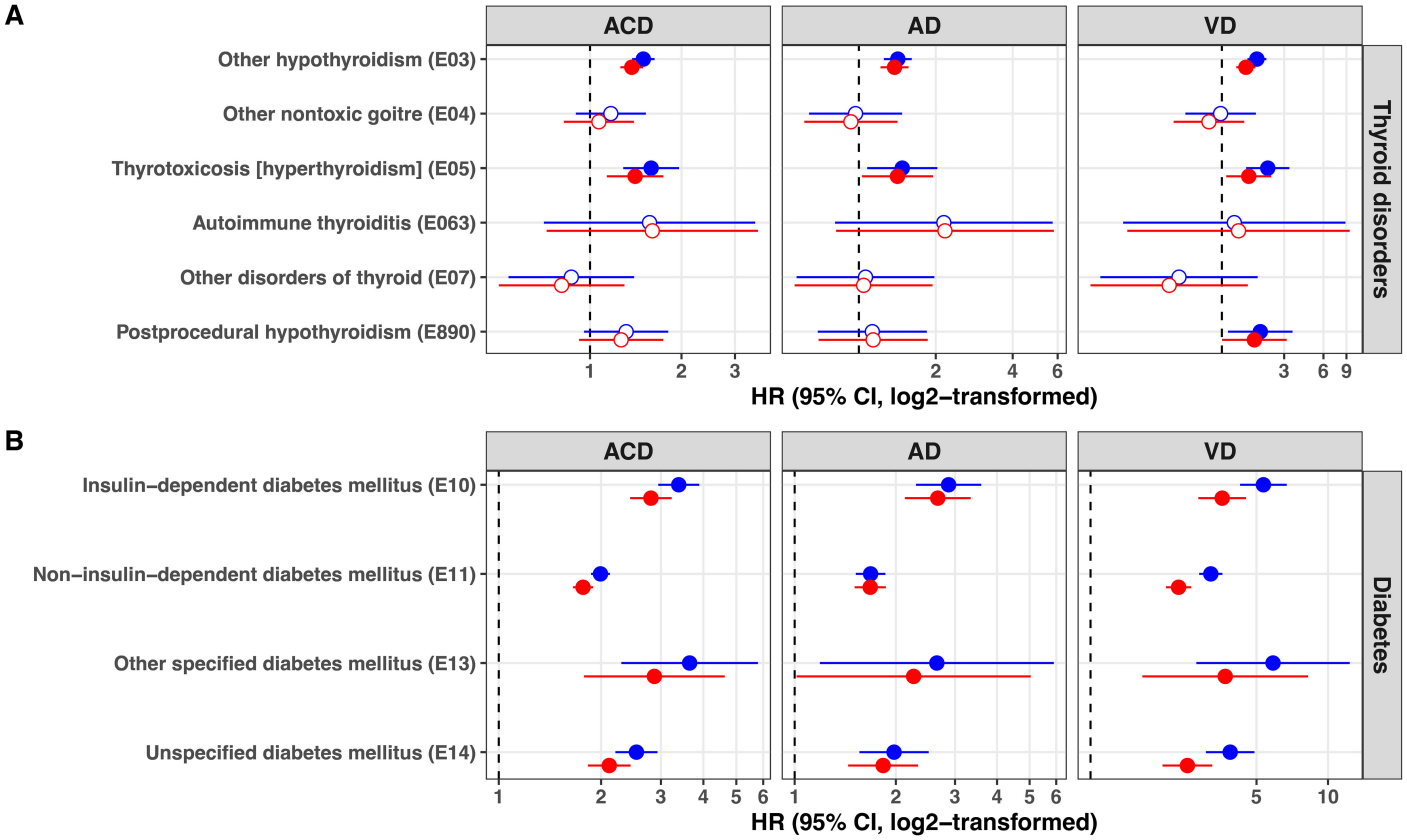
Independent associations of diabetes and thyroid disorders with risks of dementia. The Cox regression model was used for association analysis. The dots and horizontal bars mean the HR and corresponding 95% CI, respectively. Results from Model 1 and Model 2 are shown in blue and red colors, respectively. The solid and hollowed dots mean the results with (P < 0.05) and without statistical significance (P ≥ 0.05), respectively. Abbreviations: ACD, all-cause dementia; AD, Alzheimer’s disease; CI, confidence interval; HR, hazard ratio; VD, vascular dementia.

For the analyses of independent associations (**Figure 1**; **Supplementary Table 2**), we found that other hypothyroidism (E03) was associated with elevated risks of incident ACD [hazard ratio (HR) = 1.37, 95% confidence interval (CI) = 1.26 to 1.50, *P* < 0.001], AD (HR = 1.38, 95% CI = 1.22 to 1.56, *P* < 0.001), and VD (HR = 1.53, 95% CI = 1.29 to 1.81, *P* < 0.001). There were significantly positive associations between thyrotoxicosis [hyperthyroidism] (E05) and ACD/AD/VD, and between postprocedural hypothyroidism (E890) and VD. No significant results were obtained for other nontoxic goitre (E04), autoimmune thyroiditis (E063), or other disorders of thyroid (E07). Increased risks of ACD/AD/VD were found to be associated with all types of diabetes (E10, E11, E13, and E14).

In consideration of multiple comparisons, all the associations were robust except that associations between thyrotoxicosis [hyperthyroidism] (E05) and risks of AD and VD, and between postprocedural hypothyroidism (E890) and VD became to be nonsignificant (FDR-adjusted *P* values > 0.05).

### 3.3 Combined associations of diabetes and thyroid disorders with incident dementia

**Figure 2:**
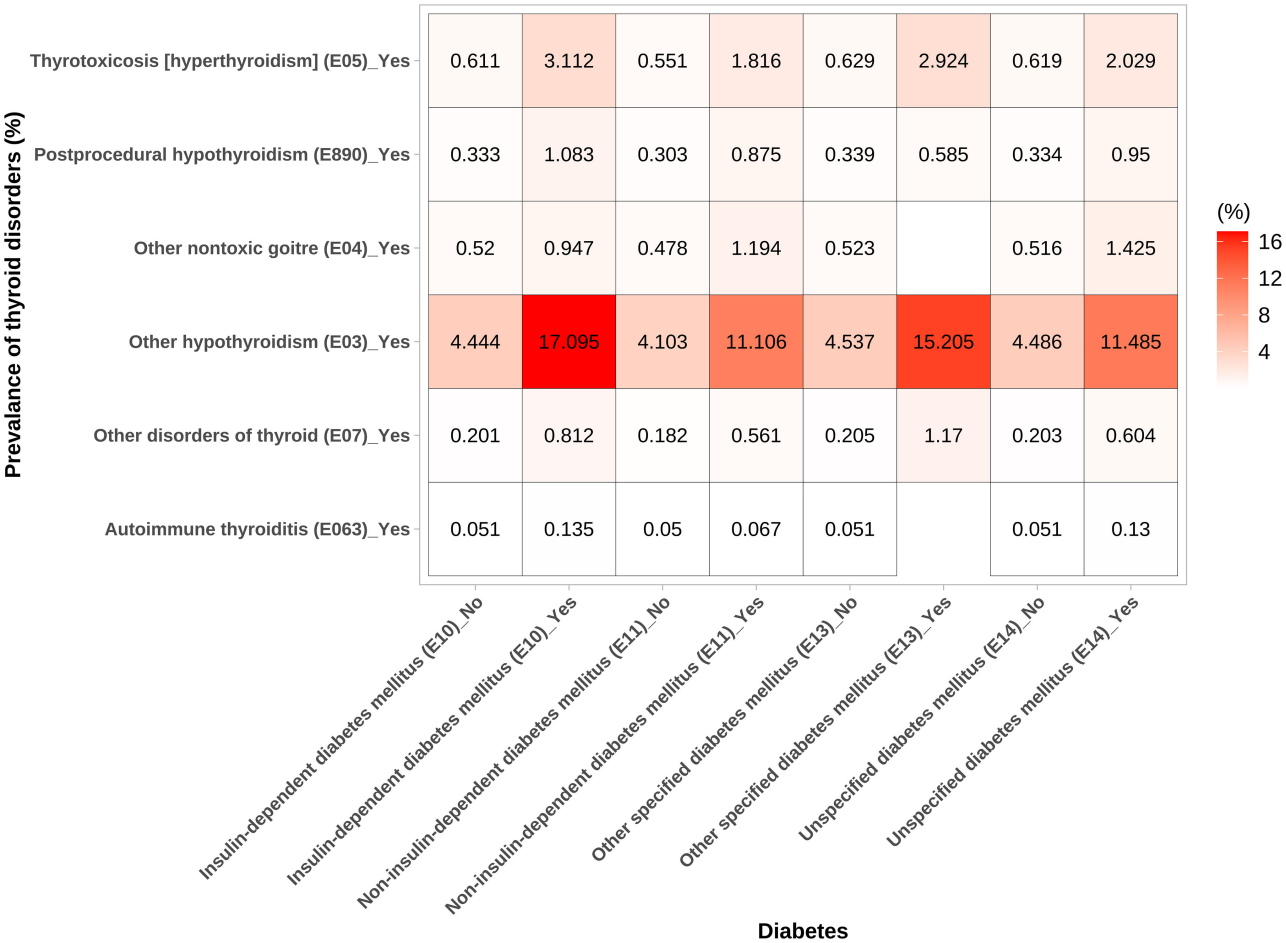
Prevalence of thyroid disorders by status of diabetes. Prevalence of thyroid disorders was separately estimated among the participants with and without diabetes. The prevalence (%) of thyroid disorders is shown in the boxes, with darker colors indicating higher values.

Patients with diabetes had a higher prevalence of thyroid disorders compared with individuals who had no such a given type of diabetes (**Figure 2**), supporting the close relationships between diabetes and thyroid disorders, especially for the hypothyroidism. As shown in **Table 2** and **Supplementary Figure 2**, the HR (95% CI) of developing an incident ACD was 1.34 (1.21 to 1.48, *P* < 0.001) for persons with only thyroid disorders, 1.76 (1.64 to 1.89, *P* < 0.001) for those with only diabetes, and 2.14 (1.82 to 2.53, *P* < 0.001) for those with both as compared to those with neither (*P* _trend_ < 0.001). Higher risks of ACD were also found for the combinations of hypothyroidism and diabetes and combinations of hyperthyroidism and diabetes. Similar results were found for the risks of AD (**Supplementary Table 3**; **Supplementary Figure 2**) and VD (**Supplementary Table 4**; **Supplementary Figure 2**).

**Table 2:** Combined associations of diabetes and thyroid disorders with incident ACD.

		Model 1 ^a^		Model 2 ^b^	
Disease status ^c^	Number of events/person-years	HR (95% CI)	*P* value	HR (95% CI)	*P* value
Hypothyroidism/diabetes					
−/−	6676/3326492	Ref (1)		Ref (1)	
+/−	432/140834	1.41 (1.28, 1.56)	< 0.001	1.34 (1.21, 1.48)	< 0.001
−/+	1004/208491	1.96 (1.83, 2.10)	< 0.001	1.76 (1.64, 1.89)	< 0.001
+/+	151/26327	2.51 (2.14, 2.95)	< 0.001	2.14 (1.82, 2.53)	< 0.001
*P*_trend_			< 0.001		< 0.001
Hyperthyroidism/diabetes					
−/−	7047/3448653	Ref (1)		Ref (1)	
+/−	61/18673	1.50 (1.16, 1.93)	0.002	1.41 (1.10, 1.82)	0.008
−/+	1129/230510	1.98 (1.86, 2.11)	< 0.001	1.77 (1.65, 1.89)	< 0.001
+/+	26/4308	2.52 (1.71, 3.70)	< 0.001	1.93 (1.30, 2.87)	0.001
*P*_trend_			< 0.001		< 0.001
Thyroid disorder/diabetes					
−/−	6651/3317229	Ref (1)		Ref (1)	
+/−	457/150096	1.41 (1.28, 1.55)	< 0.001	1.33 (1.21, 1.47)	< 0.001
−/+	993/206815	1.95 (1.83, 2.09)	< 0.001	1.76 (1.64, 1.89)	< 0.001
+/+	162/28003	2.55 (2.18, 2.98)	< 0.001	2.16 (1.84, 2.53)	< 0.001
*P*_trend_			< 0.001		< 0.001

^a^ The Cox regression model was used for association analysis. Model 1 was adjusted for age, sex, race, and education.

^b^ Model 2 was adjusted for covariates in Model 1 plus smoking, drinking, diet, physical activity, category of body mass index, allele status of *APOE*-ϵ4, and self-reported histories of heart diseases, stroke, hypertension, and emphysema/chronic bronchitis.

^c^ Hypothyroidism and hyperthyroidism were defined as the occurrence of other hypothyroidism (E03) and thyrotoxicosis [hyperthyroidism] (E05), respectively. Thyroid disorders were defined as the occurrence of hypothyroidism or hyperthyroidism. Diabetes was defined as the occurrence of any type of diabetes (E10, E11, E13, or E14).

ACD, all-cause dementia; CI, confidence interval; HR, hazard ratio; -, negative +, positive.

Besides, we found that the relative excess risk due to interaction (RERI), attributable proportion due to interaction (AP), and synergy index (SI) of diabetes and thyroid disorders were not statistically significant, suggesting their additive effects on the incidence of dementia (**Supplementary Table 5**).

### 3.4 Associations of cumulative years of diabetes and thyroid disorders with incident dementia

Compared with participants with no history of thyroid disorders, those with cumulative years of thyroid disorders at > 0 and ≤ 5 years (HR = 1.35, 95% CI = 1.18 to 1.54, *P* < 0.001), ≥ 5 and < 10 years (HR = 1.42, 95% CI = 1.26 to 1.61, *P* < 0.001), and ≥ 10 years (HR = 1.33, 95% CI = 1.10 to 1.60, *P* = 0.003) had higher risks of ACD (**Table 3**; **Supplementary Figure 3**). An increase of one cumulative year in thyroid disorders was related to an increased ACD risk of 3%. The association between cumulative years of diabetes and ACD seems to be stronger than that of thyroid disorders. We also found positive associations of cumulative years of thyroid disorders and diabetes with risks of AD (**Supplementary Table 6**; **Supplementary Figure 3**) and VD (**Supplementary Table 7**; **Supplementary Figure 3**).

**Table 3:** Associations of cumulative years of diabetes and thyroid disorders with incident ACD.

		Model 1 ^a^		Model 2 ^b^	
Cumulative years ^c^	Number of events/person-years	HR (95% CI)	*P* value	HR (95% CI)	*P* value
Thyroid disorders					
Categorical					
0	7644/3524044	Ref (1)		Ref (1)	
> 0 and ≤ 5	231/67772	1.43 (1.25, 1.63)	< 0.001	1.35 (1.18, 1.54)	< 0.001
≥ 5 and < 10	272/75049	1.59 (1.41, 1.80)	< 0.001	1.42 (1.26, 1.61)	< 0.001
≥ 10	116/35278	1.46 (1.22, 1.76)	0.002	1.33 (1.10, 1.60)	0.003
Continuous		1.04 (1.03, 1.06)	< 0.001	1.03 (1.02, 1.04)	< 0.001
Diabetes					
Categorical					
0	7108/3467326	Ref (1)		Ref (1)	
> 0 and ≤ 5	397/92469	1.71 (1.55, 1.89)	< 0.001	1.59 (1.43, 1.77)	< 0.001
≥ 5 and < 10	459/91340	2.01 (1.82, 2.20)	< 0.001	1.75 (1.59, 1.93)	< 0.001
≥ 10	299/51010	2.48 (2.20, 2.78)	< 0.001	2.12 (1.88, 2.39)	< 0.001
Continuous		1.07 (1.07, 1.08)	< 0.001	1.06 (1.05, 1.07)	< 0.001
Sum of thyroid disorders and diabetes					
Categorical					
0	6651/3317229	Ref (1)		Ref (1)	
> 0 and ≤ 5	531/142809	1.55 (1.42, 1.70)	< 0.001	1.47 (1.34, 1.61)	< 0.001
≥ 5 and < 10	620/149745	1.79 (1.65, 1.94)	< 0.001	1.60 (1.47, 1.74)	< 0.001
≥ 10	461/92361	2.21 (2.01, 2.43)	< 0.001	1.93 (1.75, 2.13)	< 0.001
Continuous		1.06 (1.05, 1.06)	< 0.001	1.05 (1.04, 1.05)	< 0.001

^a^ The Cox regression model was used for association analysis. Model 1 was adjusted for age, sex, race, and education.

^b^ Model 2 was adjusted for covariates in Model 1 plus smoking, drinking, diet, physical activity, category of body mass index, allele status of *APOE*-ϵ4, and self-reported histories of heart diseases, stroke, hypertension, and emphysema/chronic bronchitis.

^c^ Thyroid disorders were defined as the occurrence of other hypothyroidism (E03), postprocedural hypothyroidism (E890), or thyrotoxicosis [hyperthyroidism] (E05). Diabetes was defined as the occurrence of any type of diabetes (E10, E11, E13, or E14).

Abbreviations: ACD, all-cause dementia; CI, confidence interval; HR, hazard ratio.

## 4 Discussion

Based on a prospective cohort study with a large sample size and a long time of follow-up, we comprehensively analyzed associations of different types of diabetes and thyroid disorders and their combinations with risks of incident dementia. The data indicated that individuals with some types of diabetes and thyroid disorders were more likely to develop an incident ACD, AD, or VD. Besides, combined effects of diabetes and thyroid disorders on increased risks of dementia were found. More cumulative time of diabetes and thyroid disorders were related to a higher incidence of dementia. Given the usual concurrence of diabetes and thyroid disorders, as well as their attributes of potentially modifiable risks factor for dementia, our findings highlighted the importance of monitoring and maintenance of pancreatic and thyroid functions simultaneously as early as possible to protect against dementia in older adults.

Data of this study suggested positive associations between different types of diabetes and incident dementia. In line with our findings, type 2 diabetes has been linked to about 60% greater risk for incident dementia (22) and changes in brain structure (23). Since type 2 diabetes accounts for about 90-95% and type 1 diabetes for 2-5% of all cases of diabetes (24), most of the previous studies focused on the associations of type 2 diabetes and mixed diabetes with neurodegenerative disorders (8, 22, 23, 25), and fewer for the type 1 diabetes (9, 10, 26). A recent report suggested that cognitive performance declined with ageing in older adults with type 1 diabetes, especially in those at higher HbA1c levels (26). Type-1-diabetes-associated dementia risks have also been reported in other cohort studies (9, 10).

Our findings indicated higher associations of type 1 diabetes than type 2 diabetes with risks of ACD, which was similar to results from other cohort studies conducted in the UK (9) and Asian region (10). However, mechanisms involved in the type-specific associations are unclear. It has been reported that younger age at onset of diabetes is related to increased risks of incident dementia (27). Type 1 diabetes always occurs at childhood or early adult and type 2 diabetes at midlife or late life (24). As a result, stronger associations of type 1 diabetes with dementia might be induced by the longer duration of exposure. We also found that participants with other types of diabetes (ICD-10: E13 and E14) had higher risks of dementia. To the best of our knowledge, associations of other types of diabetes and incident ACD, AD, and VD have not been comprehensively examined previously.

Increased risks of dementia were found in participants with hypothyroidism or hyperthyroidism in this study. Inconsistent results have been reported on this issue. Neither hypothyroidism nor hyperthyroidism was associated with cognitive function, cognitive decline, or dementia in a previous multicohort study (13). Results from two meta-analysis studies also showed nonsignificant associations between hypothyroidism and cognitive dysfunction (28, 29), but significantly positive associations between hyperthyroidism and dementia (29). A Mendelian randomisation analysis showed causal associations between normal range levels of thyroid-stimulating hormone (TSH), a critical biomarker for the diagnosis of thyroid disorder, and decreased risks of AD (30). Another meta-analysis found a U-shaped relationship between TSH levels and risks of dementia (31), implying the complicated effects of TSH on dementia. Different results from these studies could be explained, to some extent, with the different criteria of diagnoses of thyroid disorders (28, 29). Qualified assessment on the thyroid function should be applied in the future for the standardization and parallel comparison of results from different studies.

The autoimmune thyroiditis showed no significant associations with dementia in this study. Consistently, individuals with the presence of anti-TPO antibody, a marker of autoimmune thyroid disease, had no significantly increased risks of ACD in a previous cohort study (17). However, thyroiditis-associated risks of AD have been reported in a cross-sectional study (32). Another cohort study showed higher risks of AD among cases with Hashimoto’s thyroiditis and other autoimmune disorders, but very limited covariates (age, sex, and comorbidities) were adjusted (33). Associations between postprocedural hypothyroidism and dementia have not been evaluated previously. Our results of postprocedural hypothyroidism should be explained with caution because its positive associations with VD risks became nonsignificant after the consideration of multiple comparisons.

For the first time, we found that combinations of diabetes and thyroid disorders were associated with increased risks of dementia. Besides, interactions of the two diseases on dementia were not significant, implying their additive impact of developing dementia. In line with our findings, a national population-based cohort study in Denmark has reported that the combined effects of diabetes and depression (a health status linked to thyroid dysfunction (5)) on ACD were larger than the sum of the two individual diseases (34). Besides, inflammation is closely related to thyroid function (35), combined associations of inflammation and hyperglycemia with poorer cognition were found in a cross-sectional study (36). Due to the close relationships between thyroid disorders and diabetes and their combined effects, the comorbidity of the two diseases should be given attention to expand strategies of dementia prevention.

Cumulative years of diabetes and thyroid disorders were associated higher risks of dementia in this study. Our findings were supported by several epidemiological studies (9, 15, 27). Patients with a younger age of diabetes onset had higher risks of developing an incident dementia in cohort studies (9, 27). Besides, hypothyroidism-associated dementia risks were higher in the younger patients (< 56 years old) of hypothyroidism compared with older patients (≥ 56 years old) in a cohort study of Danish population (15). However, the odds ratio of dementia associated with hypothyroidism was significant in individuals aged ≥ 65 years but not in those aged < 65 years in a cross-sectional study of Asians (14). Take diabetes for an example, associations between the onset age of diabetes and dementia risks were usually analyzed among a part of the participants because the onset age could not be estimated among individuals who were free of diabetes at baseline ^22^. The method of cumulative years of diabetes could be conducted among all participants which might be useful to enlarge the sample size.

Limitations in the present study needed to be noticed. First, some types of diabetes and thyroid disorders which had patient numbers less than 100 were not analyzed. Diabetes and thyroid disorders are often managed in primary care; using only hospital inpatient diagnoses likely misses milder or well-controlled cases. Individuals with diabetes or thyroid disorders may have more frequent healthcare contacts, increasing the chance of dementia diagnosis and an overestimated association of diabetes and thyroid disease with dementia risks. Second, more than 20,000 participants were excluded due to missing data on covariates which might induce biased estimations. Third, although fourteen covariates were adjusted for association analysis, other potential confounders which had not been collected in the UK Biobank study were not adjusted. Fourth, the present study used the history of diabetes and thyroid disorders as exposures without the consideration of corresponding medical treatments or medication uses during the follow-up. Fifth, the underline biological mechanisms linking diabetes or thyroid disorders to dementia have not been elucidated. Sixth, majority of the participants were White people which precluded the generalization of our findings to other population. Besides, the analysis of cumulative years uses disease duration from first diagnosis to baseline, not considering incident disease during follow-up. Participants who develop diabetes or thyroid disorders after baseline are treated as unexposed, which may lead to misclassification and dilute associations.

## 5 Conclusion

Data in the present study suggested that diabetes and thyroid disorders, as well as their combinations, were positively associated with increased risks of dementia among adults aged 55 years and older. Routine monitoring of pancreatic and thyroid functions is proposed for dementia prevention in older adults. Further studies are warranted to understand the mechanisms underlying the associations.

## Data Availability

The data analyzed in this study is subject to the following licenses/restrictions: The UK Biobank dataset is not fully publicly available. Access is restricted to registered researchers who submit a formal project application, obtain ethical approval and are granted official access permission by the UK Biobank. Individual-level raw data cannot be shared, uploaded or published in the article or supplementary materials due to participant privacy and data governance regulations. Requests to access these datasets should be directed to UK Biobank, https://www.ukbiobank.ac.uk/register-apply.
