# Supplementary material for: Independent and combined associations of diabetes and thyroid disorders with risks of incident dementia
Source: Front Endocrinol (Lausanne). 2026 Jul 24;17:1874555. doi: 10.3389/fendo.2026.1874555 (PMC13447225; doi:10.3389/fendo.2026.1874555)
Supplement: Supplementary file 1 [file SupplementaryFile1.docx]

**Supplementary materials**

**Figure S1.** Flow chart of participant selection.

**Table S1**. Data sources for the assessment on diabetes, thyroid disorders, and dementia.

**Table S2.** Independent associations of diabetes and thyroid disorders with risks of dementia.

**Figure S2.** Incident dementia by diabetes and thyroid disorders.

**Table S3.** Combined associations of diabetes and thyroid disorders with incident AD.

**Table S4.** Combined associations of diabetes and thyroid disorders with incident VD.

**Table S5.** Interactions between diabetes and thyroid disorders on risks of dementia.

**Table S6.** Associations of cumulative years of diabetes and thyroid disorders with incident AD.

**Table S7.** Associations of cumulative years of diabetes and thyroid disorders with incident VD.

**Figure S3.** Associations of cumulative years of diabetes and thyroid disorders with risks of dementia.

**Figure S1.** Flow chart of participant selection.

| **Table S1**. Data sources for the assessment on diabetes, thyroid disorders, and dementia. | | | | |
| --- | --- | --- | --- | --- |
| Disease category | Diseases | ICD-10 code ^a^ | Category ID in the UK Biobank | Field ID in the UK Biobank |
| Diabetes | Insulin-dependent diabetes mellitus | E10 | 2002 | 41270, 41280 |
|  | Non-insulin-dependent diabetes mellitus | E11 | 2002 | 41270, 41280 |
|  | Malnutrition-related diabetes mellitus ^b^ | E12 | 2002 | 41270, 41280 |
|  | Other specified diabetes mellitus | E13 | 2002 | 41270, 41280 |
|  | Unspecified diabetes mellitus | E14 | 2002 | 41270, 41280 |
|  | Postprocedural hypoinsulinaemia ^b^ | E891 | 2002 | 41270, 41280 |
| Thyroid disorders | Congenital iodine-deficiency syndrome ^b^ | E00 | 2002 | 41270, 41280 |
|  | Iodine-deficiency-related thyroid disorders and allied conditions ^b^ | E01 | 2002 | 41270, 41280 |
|  | Subclinical iodine-deficiency hypothyroidism ^b^ | E02 | 2002 | 41270, 41280 |
|  | Other hypothyroidism | E03 | 2002 | 41270, 41280 |
|  | Other nontoxic goitre | E04 | 2002 | 41270, 41280 |
|  | Thyrotoxicosis [hyperthyroidism] | E05 | 2002 | 41270, 41280 |
|  | Chronic thyroiditis with transient thyrotoxicosis ^b^ | E062 | 2002 | 41270, 41280 |
|  | Autoimmune thyroiditis | E063 | 2002 | 41270, 41280 |
|  | Other chronic thyroiditis ^b^ | E065 | 2002 | 41270, 41280 |
|  | Other disorders of thyroid | E07 | 2002 | 41270, 41280 |
|  | Disorders of thyroid gland in diseases classified elsewhere ^b^ | E350 | 2002 | 41270, 41280 |
|  | Postprocedural hypothyroidism | E890 | 2002 | 41270, 41280 |
| Dementia ^c^ | All-cause dementia | - | 47 | 42018 |
|  | Alzheimer’s disease | - | 47 | 42020 |
|  | Vascular dementia | - | 47 | 42022 |
|  | First occurrences of health outcomes | - | 2405, 2406 | [130836](https://biobank.ndph.ox.ac.uk/ukb/field.cgi?id=130836), [130838](https://biobank.ndph.ox.ac.uk/ukb/field.cgi?id=130838), [130840](https://biobank.ndph.ox.ac.uk/ukb/field.cgi?id=130840), [130842](https://biobank.ndph.ox.ac.uk/ukb/field.cgi?id=130842), [131036](https://biobank.ndph.ox.ac.uk/ukb/field.cgi?id=131036) |
| ^a^ ICD-10 code was extracted from the hospital inpatient data.  ^b^ These diseases were excluded due to the limited numbers of cases (< 100).  ^c^ Dementia status was ascertained through the algorithmically-defined outcomes and first occurrences of health outcomes. | | | | |

| **Table S2.** Associations between diabetes and thyroid disorders and risks of dementia. | | | | | | | | | | | |
| --- | --- | --- | --- | --- | --- | --- | --- | --- | --- | --- | --- |
|  |  |  | With diabetes (or thyroid disorders) | |  | Without diabetes (or thyroid disorders) | |  | HR for incident dementia | | |
| Dementia types | Diseases (ICD-10 code) | Model ^a^ | Number of events | Person-years |  | Number of events | Person-years |  | HR (95% CI) | *P* value | FDR-adj *P* value ^b^ |
| ACD | **Diabetes** |  |  |  |  |  |  |  |  |  |  |
|  | Insulin-dependent diabetes mellitus (E10) | Model 1 | 205 | 25651 |  | 8058 | 3676493 |  | 3.39 (2.95, 3.89) | 3.5832E-66 | 7.1664E-66 |
|  |  | Model 2 | 205 | 25651 |  | 8058 | 3676493 |  | 2.80 (2.43, 3.23) | 1.5039E-46 | 3.0078E-46 |
|  | Non-insulin-dependent diabetes mellitus (E11) | Model 1 | 1107 | 222749 |  | 7156 | 3479394 |  | 1.99 (1.87, 2.12) | 5.7456E-98 | 2.2982E-97 |
|  |  | Model 2 | 1107 | 222749 |  | 7156 | 3479394 |  | 1.77 (1.65, 1.89) | 3.0009E-60 | 1.2004E-59 |
|  | Other specified diabetes mellitus (E13) | Model 1 | 18 | 1914 |  | 8245 | 3700230 |  | 3.64 (2.29, 5.79) | 4.3706E-08 | 4.3706E-08 |
|  |  | Model 2 | 18 | 1914 |  | 8245 | 3700230 |  | 2.87 (1.78, 4.62) | 1.4575E-05 | 1.4575E-05 |
|  | Unspecified diabetes mellitus (E14) | Model 1 | 195 | 28245 |  | 8068 | 3673898 |  | 2.54 (2.20, 2.93) | 1.3814E-37 | 1.8419E-37 |
|  |  | Model 2 | 195 | 28245 |  | 8068 | 3673898 |  | 2.11 (1.83, 2.44) | 3.8118E-24 | 5.0824E-24 |
|  | **Thyroid disorders** |  |  |  |  |  |  |  |  |  |  |
|  | Other hypothyroidism (E03) | Model 1 | 583 | 167162 |  | 7680 | 3534982 |  | 1.50 (1.38, 1.63) | 1.6575E-20 | 9.9447E-20 |
|  |  | Model 2 | 583 | 167162 |  | 7680 | 3534982 |  | 1.37 (1.26, 1.50) | 6.2902E-13 | 3.7741E-12 |
|  | Other nontoxic goitre (E04) | Model 1 | 55 | 19392 |  | 8208 | 3682752 |  | 1.17 (0.90, 1.53) | 0.24428935 | 0.32362947 |
|  |  | Model 2 | 55 | 19392 |  | 8208 | 3682752 |  | 1.07 (0.82, 1.39) | 0.6206194 | 0.6206194 |
|  | Thyrotoxicosis [hyperthyroidism] (E05) | Model 1 | 87 | 22982 |  | 8176 | 3679162 |  | 1.59 (1.29, 1.96) | 1.7987E-05 | 5.3962E-05 |
|  |  | Model 2 | 87 | 22982 |  | 8176 | 3679162 |  | 1.41 (1.14, 1.74) | 0.001797 | 0.00539101 |
|  | Autoimmune thyroiditis (E063) | Model 1 | 6 | 1907 |  | 8257 | 3700237 |  | 1.57 (0.70, 3.50) | 0.26969123 | 0.32362947 |
|  |  | Model 2 | 6 | 1907 |  | 8257 | 3700237 |  | 1.60 (0.72, 3.57) | 0.24806637 | 0.37209956 |
|  | Other disorders of thyroid (E07) | Model 1 | 17 | 7666 |  | 8246 | 3694477 |  | 0.87 (0.54, 1.40) | 0.55915436 | 0.55915436 |
|  |  | Model 2 | 17 | 7666 |  | 8246 | 3694477 |  | 0.81 (0.50, 1.30) | 0.37538362 | 0.45046034 |
|  | Postprocedural hypothyroidism (E890) | Model 1 | 38 | 12482 |  | 8225 | 3689662 |  | 1.31 (0.96, 1.81) | 0.09265857 | 0.18531714 |
|  |  | Model 2 | 38 | 12482 |  | 8225 | 3689662 |  | 1.27 (0.92, 1.74) | 0.14793554 | 0.29587109 |
| AD | **Diabetes** |  |  |  |  |  |  |  |  |  |  |
|  | Insulin-dependent diabetes mellitus (E10) | Model 1 | 79 | 25987 |  | 3676 | 3687407 |  | 2.87 (2.30, 3.59) | 2.3186E-20 | 4.6372E-20 |
|  |  | Model 2 | 79 | 25987 |  | 3676 | 3687407 |  | 2.66 (2.13, 3.34) | 1.7616E-17 | 3.5232E-17 |
|  | Non-insulin-dependent diabetes mellitus (E11) | Model 1 | 434 | 224527 |  | 3321 | 3488867 |  | 1.68 (1.52, 1.86) | 9.0489E-24 | 3.6196E-23 |
|  |  | Model 2 | 434 | 224527 |  | 3321 | 3488867 |  | 1.68 (1.51, 1.87) | 2.0055E-21 | 8.0219E-21 |
|  | Other specified diabetes mellitus (E13) | Model 1 | 6 | 1928 |  | 3749 | 3711466 |  | 2.65 (1.19, 5.90) | 0.01721323 | 0.01721323 |
|  |  | Model 2 | 6 | 1928 |  | 3749 | 3711466 |  | 2.26 (1.01, 5.04) | 0.04646556 | 0.04646556 |
|  | Unspecified diabetes mellitus (E14) | Model 1 | 70 | 28595 |  | 3685 | 3684799 |  | 1.98 (1.56, 2.50) | 1.8766E-08 | 2.5021E-08 |
|  |  | Model 2 | 70 | 28595 |  | 3685 | 3684799 |  | 1.83 (1.44, 2.33) | 7.2113E-07 | 9.6151E-07 |
|  | **Thyroid disorders** |  |  |  |  |  |  |  |  |  |  |
|  | Other hypothyroidism (E03) | Model 1 | 269 | 168056 |  | 3486 | 3545338 |  | 1.42 (1.25, 1.61) | 4.2668E-08 | 2.5601E-07 |
|  |  | Model 2 | 269 | 168056 |  | 3486 | 3545338 |  | 1.38 (1.22, 1.56) | 6.1803E-07 | 3.7082E-06 |
|  | Other nontoxic goitre (E04) | Model 1 | 22 | 19462 |  | 3733 | 3693932 |  | 0.97 (0.64, 1.48) | 0.88912295 | 0.88912295 |
|  |  | Model 2 | 22 | 19462 |  | 3733 | 3693932 |  | 0.93 (0.61, 1.42) | 0.73710624 | 0.88452749 |
|  | Thyrotoxicosis [hyperthyroidism] (E05) | Model 1 | 39 | 23090 |  | 3716 | 3690304 |  | 1.48 (1.08, 2.03) | 0.01528357 | 0.0458507 |
|  |  | Model 2 | 39 | 23090 |  | 3716 | 3690304 |  | 1.42 (1.03, 1.95) | 0.033312 | 0.099936 |
|  | Autoimmune thyroiditis (E063) | Model 1 | 4 | 1909 |  | 3751 | 3711485 |  | 2.15 (0.81, 5.73) | 0.12603575 | 0.2520715 |
|  |  | Model 2 | 4 | 1909 |  | 3751 | 3711485 |  | 2.17 (0.81, 5.80) | 0.12112472 | 0.24224945 |
|  | Other disorders of thyroid (E07) | Model 1 | 10 | 7671 |  | 3745 | 3705723 |  | 1.06 (0.57, 1.98) | 0.85036364 | 0.88912295 |
|  |  | Model 2 | 10 | 7671 |  | 3745 | 3705723 |  | 1.04 (0.56, 1.94) | 0.89295627 | 0.89295627 |
|  | Postprocedural hypothyroidism (E890) | Model 1 | 16 | 12540 |  | 3739 | 3700854 |  | 1.13 (0.69, 1.85) | 0.62882709 | 0.88912295 |
|  |  | Model 2 | 16 | 12540 |  | 3739 | 3700854 |  | 1.14 (0.70, 1.86) | 0.60803936 | 0.88452749 |
| VD | **Diabetes** |  |  |  |  |  |  |  |  |  |  |
|  | Insulin-dependent diabetes mellitus (E10) | Model 1 | 79 | 25978 |  | 1814 | 3691599 |  | 5.34 (4.26, 6.70) | 1.1664E-47 | 2.3327E-47 |
|  |  | Model 2 | 79 | 25978 |  | 1814 | 3691599 |  | 3.58 (2.84, 4.52) | 3.4113E-27 | 6.8226E-27 |
|  | Non-insulin-dependent diabetes mellitus (E11) | Model 1 | 396 | 224486 |  | 1497 | 3493091 |  | 3.21 (2.87, 3.59) | 3.8796E-91 | 1.5519E-90 |
|  |  | Model 2 | 396 | 224486 |  | 1497 | 3493091 |  | 2.35 (2.08, 2.65) | 1.9708E-42 | 7.8833E-42 |
|  | Other specified diabetes mellitus (E13) | Model 1 | 7 | 1931 |  | 1886 | 3715646 |  | 5.87 (2.79, 12.34) | 3.096E-06 | 3.096E-06 |
|  |  | Model 2 | 7 | 1931 |  | 1886 | 3715646 |  | 3.69 (1.65, 8.24) | 0.00144343 | 0.00144343 |
|  | Unspecified diabetes mellitus (E14) | Model 1 | 73 | 28573 |  | 1820 | 3689004 |  | 3.87 (3.06, 4.90) | 1.8633E-29 | 2.4845E-29 |
|  |  | Model 2 | 73 | 28573 |  | 1820 | 3689004 |  | 2.56 (2.01, 3.25) | 2.208E-14 | 2.944E-14 |
|  | **Thyroid disorders** |  |  |  |  |  |  |  |  |  |  |
|  | Other hypothyroidism (E03) | Model 1 | 155 | 168324 |  | 1738 | 3549253 |  | 1.85 (1.57, 2.19) | 4.3002E-13 | 2.5801E-12 |
|  |  | Model 2 | 155 | 168324 |  | 1738 | 3549253 |  | 1.53 (1.29, 1.81) | 9.6224E-07 | 5.7734E-06 |
|  | Other nontoxic goitre (E04) | Model 1 | 10 | 19484 |  | 1883 | 3698093 |  | 0.98 (0.52, 1.82) | 0.94376344 | 0.94376344 |
|  |  | Model 2 | 10 | 19484 |  | 1883 | 3698093 |  | 0.80 (0.43, 1.48) | 0.4722649 | 0.56671788 |
|  | Thyrotoxicosis [hyperthyroidism] (E05) | Model 1 | 27 | 23133 |  | 1866 | 3694444 |  | 2.25 (1.54, 3.29) | 3.1074E-05 | 9.3223E-05 |
|  |  | Model 2 | 27 | 23133 |  | 1866 | 3694444 |  | 1.61 (1.08, 2.39) | 0.01926684 | 0.05780053 |
|  | Autoimmune thyroiditis (E063) | Model 1 | 1 | 1915 |  | 1892 | 3715663 |  | 1.24 (0.18, 8.85) | 0.82669303 | 0.94376344 |
|  |  | Model 2 | 1 | 1915 |  | 1892 | 3715663 |  | 1.34 (0.19, 9.53) | 0.76940859 | 0.76940859 |
|  | Other disorders of thyroid (E07) | Model 1 | 2 | 7699 |  | 1891 | 3709878 |  | 0.47 (0.12, 1.88) | 0.28530039 | 0.42795058 |
|  |  | Model 2 | 2 | 7699 |  | 1891 | 3709878 |  | 0.39 (0.10, 1.58) | 0.1892327 | 0.28384905 |
|  | Postprocedural hypothyroidism (E890) | Model 1 | 12 | 12545 |  | 1881 | 3705033 |  | 1.97 (1.11, 3.48) | 0.01957889 | 0.03915779 |
|  |  | Model 2 | 12 | 12545 |  | 1881 | 3705033 |  | 1.77 (1.00, 3.13) | 0.04830865 | 0.0966173 |
| ^a^ The Cox regression model was used for association analysis. Model 1 was adjusted for age, sex, race, and education. Model 2 was adjusted for covariates in Model 1 plus smoking, drinking, diet, physical activity, category of body mass index, allele status of *APOE*-ε4, and self-reported histories of heart diseases, stroke, hypertension, and emphysema/chronic bronchitis.  ^b^ *P* values were adjusted with the method of false discovery rate to account for multiple comparisons.  Abbreviations: ACD, all-cause dementia; AD, Alzheimer’s disease; CI, confidence interval; FDR, false discovery rate; HR, hazard ratio; VD, vascular dementia. | | | | | | | | | | | |

**Figure S2.** Incident dementia by diabetes and thyroid disorders. Kaplan-Meier curves show the survival probabilities of incident dementia by diabetes and thyroid disorders (log-rank test; all *P* < 0.001). Abbreviations: ACD, all-cause dementia; AD, Alzheimer’s disease; VD, vascular dementia; hypo, hypothyroidism; hyper, hyperthyroidism; thy, thyroid disorders; diab, diabetes; -, negative; +, positive.

| **Table S3.** Combined associations of diabetes and thyroid disorders with incident AD. | | | | | | |
| --- | --- | --- | --- | --- | --- | --- |
|  |  | Model 1 ^a^ |  |  | Model 2 ^b^ |  |
| Disease status ^c^ | Number of events/ Person-years | HR (95% CI) | *P* value |  | HR (95% CI) | *P* value |
| Hypothyroidism / diabetes |  |  |  |  |  |  |
| $-/-$ | 3103/3335247 | Ref (1) |  |  | Ref (1) |  |
| $+/-$ | 200/141489 | 1.31 (1.13, 1.51) | < 0.001 |  | 1.29 (1.12, 1.50) | < 0.001 |
| $-/+$ | 383/210091 | 1.61 (1.45, 1.80) | < 0.001 |  | 1.62 (1.44, 1.81) | < 0.001 |
| $+/+$ | 69/26568 | 2.33 (1.83, 2.96) | < 0.001 |  | 2.30 (1.80, 2.93) | < 0.001 |
| *P* _trend_ |  |  | < 0.001 |  |  | < 0.001 |
| Hyperthyroidism / diabetes |  |  |  |  |  |  |
| $-/-$ | 3274/3457991 | Ref (1) |  |  | Ref (1) |  |
| $+/-$ | 29/18744 | 1.44 (1.00, 2.08) | 0.049 |  | 1.41 (0.97, 2.05) | 0.068 |
| $-/+$ | 442/232313 | 1.67 (1.51, 1.85) | < 0.001 |  | 1.66 (1.50, 1.85) | < 0.001 |
| $+/+$ | 10/4345 | 1.98 (1.06, 3.68) | 0.032 |  | 1.88 (1.01, 3.50) | 0.047 |
| *P* _trend_ |  |  | < 0.001 |  |  | < 0.001 |
| Thyroid disorders / diabetes |  |  |  |  |  |  |
| $-/-$ | 3089/3325971 | Ref (1) |  |  | Ref (1) |  |
| $+/-$ | 214/150765 | 1.33 (1.15, 1.53) | 0.001 |  | 1.31 (1.14, 1.50) | 0.001 |
| $-/+$ | 380/208397 | 1.61 (1.45, 1.80) | < 0.001 |  | 1.62 (1.44, 1.81) | < 0.001 |
| $+/+$ | 72/28261 | 2.30 (1.82, 2.91) | < 0.001 |  | 2.27 (1.79, 2.88) | < 0.001 |
| *P* _trend_ |  |  | < 0.001 |  |  | < 0.001 |
| ^a^ Model 1 was adjusted for age, sex, race, and education.  ^b^ Model 2 was adjusted for covariates in Model 1 plus smoking, drinking, diet, physical activity, category of body mass index, allele status of *APOE*-ε4, and self-reported histories of heart diseases, stroke, hypertension, and emphysema/chronic bronchitis.  ^c^ Hypothyroidism and hyperthyroidism were defined as the occurrence of other hypothyroidism (E03) and thyrotoxicosis [hyperthyroidism] (E05), respectively. Thyroid disorders were defined as the occurrence of hypothyroidism or hyperthyroidism. Diabetes was defined as the occurrence of any type of diabetes (E10, E11, E13, or E14).  Abbreviations: AD, Alzheimer’s disease; CI, confidence interval; HR, hazard ratio; -, negative +, positive. | | | | | | |

| **Table S4.** Combined associations of diabetes and thyroid disorders with incident VD. | | | | | | |
| --- | --- | --- | --- | --- | --- | --- |
|  |  | Model 1 ^a^ |  |  | Model 2 ^b^ |  |
| Disease status ^c^ | Number of events/ Person-years | HR (95% CI) | *P* value |  | HR (95% CI) | *P* value |
| Hypothyroidism / diabetes |  |  |  |  |  |  |
| $-/-$ | 1385/3339241 | Ref (1) |  |  | Ref (1) |  |
| $+/-$ | 98/141702 | 1.62 (1.32, 1.99) | < 0.001 |  | 1.46 (1.18, 1.79) | < 0.001 |
| $-/+$ | 353/210013 | 3.11 (2.77, 3.51) | < 0.001 |  | 2.33 (2.05, 2.65) | < 0.001 |
| $+/+$ | 57/26622 | 4.56 (3.49, 5.95) | < 0.001 |  | 3.01 (2.28, 3.97) | < 0.001 |
| *P* _trend_ |  |  | < 0.001 |  |  | < 0.001 |
| Hyperthyroidism / diabetes |  |  |  |  |  |  |
| $-/-$ | 1464/3462154 | Ref (1) |  |  | Ref (1) |  |
| $+/-$ | 19/18789 | 2.34 (1.48, 3.68) | < 0.001 |  | 1.95 (1.22, 3.10) | 0.005 |
| $-/+$ | 402/232290 | 3.20 (2.86, 3.58) | < 0.001 |  | 2.37 (2.10, 2.68) | < 0.001 |
| $+/+$ | 8/4344 | 3.74 (1.86, 7.49) | < 0.001 |  | 1.89 (0.90, 3.99) | 0.095 |
| *P* _trend_ |  |  | < 0.001 |  |  | < 0.001 |
| Thyroid disorders / diabetes |  |  |  |  |  |  |
| $-/-$ | 1377/3329929 | Ref (1) |  |  | Ref (1) |  |
| $+/-$ | 106/151015 | 1.66 (1.36, 2.02) | < 0.001 |  | 1.48 (1.21, 1.81) | 0.001 |
| $-/+$ | 350/208329 | 3.12 (2.77, 3.51) | < 0.001 |  | 2.34 (2.06, 2.66) | < 0.001 |
| $+/+$ | 60/28305 | 4.55 (3.51, 5.90) | < 0.001 |  | 2.94 (2.24, 3.87) | < 0.001 |
| *P* _trend_ |  |  | < 0.001 |  |  | < 0.001 |
| ^a^ Model 1 was adjusted for age, sex, race, and education.  ^b^ Model 2 was adjusted for covariates in Model 1 plus smoking, drinking, diet, physical activity, category of body mass index, allele status of *APOE*-ε4, and self-reported histories of heart diseases, stroke, hypertension, and emphysema/chronic bronchitis.  ^c^ Hypothyroidism and hyperthyroidism were defined as the occurrence of other hypothyroidism (E03) and thyrotoxicosis [hyperthyroidism] (E05), respectively. Thyroid disorders were defined as the occurrence of hypothyroidism or hyperthyroidism. Diabetes was defined as the occurrence of any type of diabetes (E10, E11, E13, or E14).  Abbreviations: CI, confidence interval; HR, hazard ratio; VD, vascular dementia; -, negative +, positive. | | | | | | |

| **Table S5.** Interactions between diabetes and thyroid disorders on risks of dementia. | | | | | | | | | | | |
| --- | --- | --- | --- | --- | --- | --- | --- | --- | --- | --- | --- |
|  |  |  |  | RERI |  |  | AP |  |  | SI |  |
| Thyroid disorders ^a^ | Diabetes ^a^ | Dementia | Models ^b^ | Estimation (95% CI) | *P* value |  | Estimation (95% CI) | *P* value |  | Estimation (95% CI) | *P* value |
| Overall thyroid disorders | Diabetes | ACD | Model 1 | 0.18 (-0.25, 0.62) | 0.201 |  | 0.07 (-0.09, 0.23) | 0.186 |  | 1.14 (0.85, 1.51) | 0.192 |
|  |  | ACD | Model 2 | -0.13 (-0.45, 0.19) | 0.783 |  | -0.08 (-0.29, 0.13) | 0.232 |  | 0.83 (0.51, 1.36) | 0.766 |
|  |  | AD | Model 1 | 0.36 (-0.23, 0.94) | 0.115 |  | 0.16 (-0.07, 0.38) | 0.085 |  | 1.38 (0.85, 2.24) | 0.097 |
|  |  | AD | Model 2 | 0.13 (-0.37, 0.64) | 0.303 |  | 0.08 (-0.19, 0.34) | 0.290 |  | 1.21 (0.62, 2.38) | 0.290 |
|  |  | VD | Model 1 | 0.78 (-0.46, 2.02) | 0.110 |  | 0.17 (-0.06, 0.40) | 0.075 |  | 1.28 (0.89, 1.84) | 0.092 |
|  |  | VD | Model 2 | -0.34 (-1.06, 0.37) | 0.828 |  | -0.17 (-0.58, 0.24) | 0.205 |  | 0.75 (0.38, 1.48) | 0.799 |
| Hypothyroidism | Diabetes | ACD | Model 1 | 0.14 (-0.30, 0.58) | 0.263 |  | 0.06 (-0.11, 0.22) | 0.253 |  | 1.10 (0.82, 1.49) | 0.257 |
|  |  | ACD | Model 2 | 0.05 (-0.34, 0.43) | 0.406 |  | 0.02 (-0.15, 0.20) | 0.405 |  | 1.04 (0.74, 1.46) | 0.405 |
|  |  | AD | Model 1 | 0.40 (-0.20, 1.00) | 0.094 |  | 0.17 (-0.05, 0.40) | 0.063 |  | 1.44 (0.88, 2.35) | 0.075 |
|  |  | AD | Model 2 | 0.39 (-0.21, 0.98) | 0.100 |  | 0.17 (-0.05, 0.39) | 0.068 |  | 1.43 (0.87, 2.35) | 0.080 |
|  |  | VD | Model 1 | 0.83 (-0.44, 2.10) | 0.101 |  | 0.18 (-0.05, 0.42) | 0.066 |  | 1.30 (0.90, 1.89) | 0.083 |
|  |  | VD | Model 2 | 0.21 (-0.67, 1.10) | 0.318 |  | 0.07 (-0.21, 0.35) | 0.307 |  | 1.12 (0.71, 1.76) | 0.312 |
| Hyperthyroidism | Diabetes | ACD | Model 1 | 0.04 (-1.01, 1.08) | 0.470 |  | 0.02 (-0.39, 0.43) | 0.470 |  | 1.03 (0.51, 2.05) | 0.470 |
|  |  | ACD | Model 2 | -0.25 (-1.09, 0.60) | 0.716 |  | -0.13 (-0.61, 0.35) | 0.302 |  | 0.79 (0.33, 1.89) | 0.701 |
|  |  | AD | Model 1 | -0.14 (-1.48, 1.21) | 0.580 |  | -0.07 (-0.79, 0.65) | 0.425 |  | 0.88 (0.23, 3.37) | 0.576 |
|  |  | AD | Model 2 | -0.20 (-1.49, 1.09) | 0.620 |  | -0.11 (-0.85, 0.64) | 0.390 |  | 0.81 (0.20, 3.37) | 0.612 |
|  |  | VD | Model 1 | -0.80 (-3.61, 2.02) | 0.711 |  | -0.21 (-1.10, 0.68) | 0.319 |  | 0.77 (0.29, 2.10) | 0.693 |
|  |  | VD | Model 2 | -1.43 (-3.11, 0.26) | 0.952 |  | -0.76 (-2.15, 0.64) | 0.144 |  | 0.38 (0.08, 1.95) | 0.876 |
| ^a^ Hypothyroidism and hyperthyroidism were defined as the occurrence of other hypothyroidism (E03) and thyrotoxicosis [hyperthyroidism] (E05), respectively. Thyroid disorders were defined as the occurrence of hypothyroidism or hyperthyroidism. Diabetes was defined as the occurrence of any type of diabetes (E10, E11, E13, or E14).  ^b^ The Cox regression model was used for association analysis. Model 1 was adjusted for age, sex, race, and education. Model 2 was adjusted for covariates in Model 1 plus smoking, drinking, diet, physical activity, category of body mass index, allele status of *APOE*-ε4, and self-reported histories of heart diseases, stroke, hypertension, and emphysema/chronic bronchitis.  Abbreviations: ACD, all-cause dementia; AD, Alzheimer’s disease; AP, attributable proportion due to interaction; CI, confidence interval; RERI, relative excess risk due to interaction; SI, synergy index; VD, vascular dementia. | | | | | | | | | | | |

| **Table S6.** Associations of cumulative years of diabetes and thyroid disorders with incident AD. | | | | | | |
| --- | --- | --- | --- | --- | --- | --- |
|  |  | Model 1 ^a^ |  |  | Model 2 ^b^ |  |
| Cumulative years ^c^ | Number of events/ Person-years | HR (95% CI) | *P* value |  | HR (95% CI) | *P* value |
| Thyroid disorders |  |  |  |  |  |  |
| Categorical |  |  |  |  |  |  |
| 0 | 3469/3534368 | Ref (1) |  |  | Ref (1) |  |
| > 0 and ≤ 5 | 106/68105 | 1.35 (1.11, 1.64) | 0.002 |  | 1.34 (1.10, 1.63) | 0.003 |
| ≥ 5 and < 10 | 128/75440 | 1.53 (1.29, 1.83) | < 0.001 |  | 1.46 (1.22, 1.74) | < 0.001 |
| ≥ 10 | 52/35481 | 1.36 (1.03, 1.78) | 0.030 |  | 1.31 (1.00, 1.73) | 0.052 |
| Continuous |  | 1.04 (1.02, 1.05) | < 0.001 |  | 1.03 (1.02, 1.05) | 0.001 |
| Diabetes |  |  |  |  |  |  |
| Categorical |  |  |  |  |  |  |
| 0 | 3303/3476736 | Ref (1) |  |  | Ref (1) |  |
| > 0 and ≤ 5 | 158/93098 | 1.47 (1.25, 1.73) | < 0.001 |  | 1.52 (1.29, 1.79) | < 0.001 |
| ≥ 5 and < 10 | 183/91986 | 1.72 (1.48, 1.99) | < 0.001 |  | 1.68 (1.44, 1.96) | < 0.001 |
| ≥ 10 | 111/51575 | 1.97 (1.63, 2.38) | < 0.001 |  | 1.90 (1.56, 2.30) | < 0.001 |
| Continuous |  | 1.06 (1.05, 1.07) | < 0.001 |  | 1.05 (1.04, 1.06) | < 0.001 |
| Sum of thyroid disorders and diabetes |  |  |  |  |  |  |
| Categorical |  |  |  |  |  |  |
| 0 | 3089/3325971 | Ref (1) |  |  | Ref (1) |  |
| > 0 and ≤ 5 | 223/143573 | 1.38 (1.20, 1.58) | < 0.001 |  | 1.40 (1.22, 1.61) | < 0.001 |
| ≥ 5 and < 10 | 259/150659 | 1.56 (1.38, 1.77) | < 0.001 |  | 1.52 (1.34, 1.73) | < 0.001 |
| ≥ 10 | 184/93191 | 1.84 (1.58, 2.13) | < 0.001 |  | 1.79 (1.53, 2.08) | < 0.001 |
| Continuous |  | 1.05 (1.04, 1.05) | < 0.001 |  | 1.04 (1.03, 1.05) | < 0.001 |
| ^a^ Model 1 was adjusted for age, sex, race, and education.  ^b^ Model 2 was adjusted for covariates in Model 1 plus smoking, drinking, diet, physical activity, category of body mass index, allele status of *APOE*-ε4, and self-reported histories of heart diseases, stroke, hypertension, and emphysema/chronic bronchitis.  ^c^ Thyroid disorders were defined as the occurrence of other hypothyroidism (E03) or thyrotoxicosis [hyperthyroidism] (E05). Diabetes was defined as the occurrence of any type of diabetes (E10, E11, E13, or E14).  Abbreviations: AD, Alzheimer’s disease; CI, confidence interval; HR, hazard ratio. | | | | | | |

| **Table S7.** Associations of cumulative years of diabetes and thyroid disorders with incident VD. | | | | | | |
| --- | --- | --- | --- | --- | --- | --- |
|  |  | Model 1 ^a^ |  |  | Model 2 ^b^ |  |
| Cumulative years ^c^ | Number of events/ Person-years | HR (95% CI) | *P* value |  | HR (95% CI) | *P* value |
| Thyroid disorders |  |  |  |  |  |  |
| Categorical |  |  |  |  |  |  |
| 0 | 1727/3538258 | Ref (1) |  |  | Ref (1) |  |
| > 0 and ≤ 5 | 60/68183 | 1.72 (1.33, 2.23) | < 0.001 |  | 1.49 (1.14, 1.93) | 0.003 |
| ≥ 5 and < 10 | 71/75610 | 1.95 (1.54, 2.48) | < 0.001 |  | 1.52 (1.19, 1.94) | 0.001 |
| ≥ 10 | 35/35526 | 2.06 (1.47, 2.89) | < 0.001 |  | 1.64 (1.17, 2.30) | 0.004 |
| Continuous |  | 1.07 (1.04, 1.09) | < 0.001 |  | 1.04 (1.02, 1.07) | < 0.001 |
| Diabetes |  |  |  |  |  |  |
| Categorical |  |  |  |  |  |  |
| 0 | 1483/3480943 | Ref (1) |  |  | Ref (1) |  |
| > 0 and ≤ 5 | 126/93110 | 2.46 (2.04, 2.95) | < 0.001 |  | 1.94 (1.61, 2.34) | < 0.001 |
| ≥ 5 and < 10 | 167/92045 | 3.30 (2.81, 3.88) | < 0.001 |  | 2.36 (1.99, 2.79) | < 0.001 |
| ≥ 10 | 117/51479 | 4.37 (3.61, 5.28) | < 0.001 |  | 3.05 (2.50, 3.71) | < 0.001 |
| Continuous |  | 1.11 (1.10, 1.12) | < 0.001 |  | 1.08 (1.07, 1.09) | < 0.001 |
| Sum of thyroid disorders and diabetes |  |  |  |  |  |  |
| Categorical |  |  |  |  |  |  |
| 0 | 1377/3329929 | Ref (1) |  |  | Ref (1) |  |
| > 0 and ≤ 5 | 149/143629 | 2.05 (1.73, 2.43) | < 0.001 |  | 1.74 (1.46, 2.06) | < 0.001 |
| ≥ 5 and < 10 | 198/150863 | 2.72 (2.34, 3.16) | < 0.001 |  | 2.06 (1.76, 2.41) | < 0.001 |
| ≥ 10 | 169/93156 | 3.84 (3.27, 4.51) | < 0.001 |  | 2.74 (2.32, 3.24) | < 0.001 |
| Continuous |  | 1.09 (1.08, 1.10) | < 0.001 |  | 1.07 (1.06, 1.08) | < 0.001 |
| ^a^ Model 1 was adjusted for age, sex, race, and education.  ^b^ Model 2 was adjusted for covariates in Model 1 plus smoking, drinking, diet, physical activity, category of body mass index, allele status of *APOE*-ε4, and self-reported histories of heart diseases, stroke, hypertension, and emphysema/chronic bronchitis.  ^c^ Thyroid disorders were defined as the occurrence of other hypothyroidism (E03) or thyrotoxicosis [hyperthyroidism] (E05). Diabetes was defined as the occurrence of any type of diabetes (E10, E11, E13, or E14).  Abbreviations: CI, confidence interval; HR, hazard ratio; VD, vascular disease. | | | | | | |

**Figure S3.** Associations of cumulative years of diabetes and thyroid disorders with risks of dementia. The fully adjusted Cox regression model was used for association analysis. The HR (95% CI) was plotted against cumulative years of thyroid disorders (in red), diabetes (in green), and the sum of cumulative years of thyroid disorders and diabetes (in blue). The lines and areas mean the HR and corresponding 95% CI, respectively. Thyroid disorders were defined as the occurrence of other hypothyroidism (E03) or thyrotoxicosis [hyperthyroidism] (E05). Diabetes was defined as the occurrence of any type of diabetes (E10, E11, E13, or E14). Abbreviations: ACD, all-cause dementia; AD, Alzheimer’s disease; CI, confidence interval; HR, hazard ratio; VD, vascular dementia.
